# Imaging the microanatomy of astrocyte–T-cell interactions in immune-mediated inflammation

**DOI:** 10.3389/fncel.2013.00058

**Published:** 2013-04-30

**Authors:** Carlos Barcia, Izaskun Mitxitorena, María A. Carrillo-de Sauvage, José-María Gallego, Ana Pérez-Vallés, Carlos Barcia

**Affiliations:** ^1^Department of Neurosurgery, Hospital General Universitari de ValènciaValència, Spain; ^2^Lab M2-107, Institut de Neurociències, Universitat Autònoma de Barcelona, Bellaterra, Cerdanyola del VallèsBarcelona, Spain; ^3^Départment des Sciences du Vivant, Institut d'Imagerie Biomédicale, MIRCen and CNRS, URA2210 (I2BM), Commissariat à l'Energie Atomique et aux Energies Alternatives, Fontenay-aux-RosesParis, France; ^4^Department of Pathology, Hospital General Universitari de ValènciaValència, Spain; ^5^Department de Bioquímica i de Biologia Molecular, School of Medicine, Universitat Autònoma de Barcelona, Bellaterra, Cerdanyola del VallèsBarcelona, Spain

**Keywords:** astrocyte, T-cell, immunological synapse, infiltration, cytokines, chemokines, glioma, immune response

## Abstract

The role of astrocytes in the immune-mediated inflammatory response in the brain is more prominent than previously thought. Astrocytes become reactive in response to neuro-inflammatory stimuli through multiple pathways, contributing significantly to the machinery that modifies the parenchymal environment. In particular, astrocytic signaling induces the establishment of critical relationships with infiltrating blood cells, such as lymphocytes, which is a fundamental process for an effective immune response. The interaction between astrocytes and T-cells involves complex modifications to both cell types, which undergo micro-anatomical changes and the redistribution of their binding and secretory domains. These modifications are critical for different immunological responses, such as for the effectiveness of the T-cell response, for the specific infiltration of these cells and their homing in the brain parenchyma, and for their correct apposition with antigen-presenting cells (APCs) to form immunological synapses (ISs). In this article, we review the current knowledge of the interactions between T-cells and astrocytes in the context of immune-mediated inflammation in the brain, based on the micro-anatomical imaging of these appositions by high-resolution confocal microscopy and three-dimensional rendering. The study of these dynamic interactions using detailed technical approaches contributes to understanding the function of astrocytes in inflammatory responses and paves the way for new therapeutic strategies.

## Introduction

Astrocytes are sensitive to changes in the brain parenchyma and become reactive in the presence of inflammatory stimuli, changing both their protein expression and phenotype. These cellular modifications are characterized by an apparent augmentation of cell body size, an increased number of primary and secondary branches, and an intensification of various signaling cascades, such as ATP-mediated and cytokine-mediated signaling, often resulting in the overexpression of certain proteins, such as intermediate filament proteins, cytokines, and chemokines (Buffo et al., 2010; Kang and Hebert, 2011; Molofsky et al., 2012; Sun and Jakobs, 2012). The role of these responses is not well established and might be multifaceted and different for each neuro-pathological scenario and for each brain region (Zhang and Barres, 2010; Oberheim et al., 2012). Generally, when astrocytes become reactive in response to injury or inflammation, glial fibrillary acidic protein (GFAP), as well as other intermediate filament proteins such as nestin and vimentin, become overexpressed (Pekny and Nilsson, 2005; Buffo et al., 2008, 2010). Although it is a widely used and reliable marker for astrocytes, GFAP's function remains poorly understood. Furthermore, the existence of different isoforms of GFAP and different populations of astrocytes adds further complexity to understanding the role of GFAP. It is thought that GFAP may participate in many cellular processes, such as motility, proliferation, autophagy, synaptic plasticity, neural outgrowth, myelination, injury protection, and blood brain barrier (BBB) formation (Middeldorp and Hol, 2011). Importantly, some of these tasks are intimately related to immune-mediated inflammation and seem to be crucial for adequate responses in pathological conditions, particularly for protoplasmic astrocytes, which reside in the gray matter and form the neurovascular unit (Dirnagl, 2012).

The mechanism that induces the spatial reorganization of astrocytes appears to be multifaceted (Buffo et al., 2010; Kang and Hebert, 2011). One of the most important pathways that triggers this reactivity involves cAMP, which represents a crucial player in astrocyte cell-to-cell communication (Grimaldi et al., 1999; Pascual et al., 2005). Injured cells release or leak ATP into the intercellular space, thereby activating the P2 receptors of neighboring astrocytes and initiating structural modifications. Additionally, the release of cytokines into the extracellular space, either by infiltrating blood cells or by neighboring glial cells, also contributes to astrocytic reactivity by stimulating cytokine receptors present on the astrocyte cell surface (Buffo et al., 2010; Hamby and Sofroniew, 2010).

**Figure 1:**
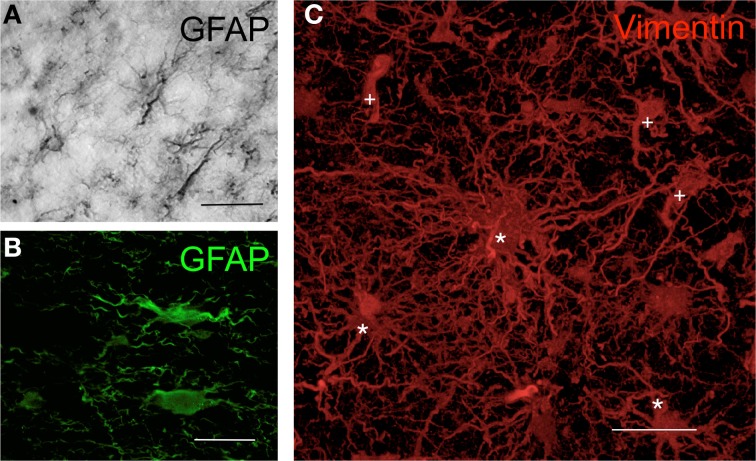
**Morphology of human astrocytes by immunohisto-chemistry. (A)** Astrocytes in the human cortex visualized by GFAP immunohistochemistry through DAB precipitation in the peritumoral area of a glioma. **(B)** Astrocytes in human cortex visualized by GFAP immunofluorescence in the peritumoral area of a glioma. **(C)** Three-dimensional transparency of vimentin^+^ cells in human glioblastoma. Cells in the tumor areas were marked using immunohistochemistry with antibodies against vimentin (red) and were scanned with high-resolution confocal microscopy. Some cells maintain the typical astrocytic star shape (^*^), whereas others show the neoplastic morphology (+) that is characteristic of astrocytomas [modified from Carrillo-de Sauvage et al. (2012)]. Scale bar: 30 μm in **(A)** and **(C)**; 25 μm in **(B)**.

Inflammatory GFAP immunoreactivity can be observed in brain injuries and in most prominent neurodegenerative disorders, such as Parkinson's disease (Forno et al., 1992), Alzheimer's disease (Diedrich et al., 1987), Huntington's disease (Galatioto, 1996), and multiple sclerosis (Darvesh et al., 2010), suggesting the involvement of glia-mediated inflammation in the process of degeneration. Particular attention should be given to gliomas, which are primary brain tumors that have astrocytic characteristics. Glioma cells originate from neural stem cells or oligodendrocyte progenitor cells (Sanai et al., 2005; Lindberg et al., 2009) but can also be derived from mature astrocytes or differentiated neurons that change their phenotype to a glioma cell-type (Friedmann-Morvinski et al., 2012). However, independently of their origin, glioma cells express high amounts of GFAP and vimentin (Herpers et al., 1986), much like reactive astrocytes in other inflammatory scenarios. Glioma cell morphology within the tumor is variable; it is difficult to define a particular shape for these cells. In addition, glioma cells are co-located with non-neoplastic resident astrocytes, which makes it even more difficult to distinguish between these two cell types (Figure 1). In experimental models, marking inoculated glioma cells with green fluorescent protein (GFP) or with similar markers makes it possible to identify glioma cells (Tatenhorst et al., 2005). In this particular model, artificially implanted GFP^+^ glioma cells adopt a unipolar morphology, with the major processes closely aligned with blood vessels (BVs) and intercalated between endothelial cells and resident GFAP^+^ protoplasmic astrocytes (Farin et al., 2006). This evidence suggests that similar intercellular apposition between resident astrocytes and glioma cells may occur in human glioma. In addition, glioma cells are also in constant mitosis, which entails frequent morphological changes and dramatic modifications of the intercellular space, together with the breaking of the intercellular matrix (Ziu et al., 2006). Together, these features of the astrocytic reactivity of glioma cells are important contributors to glia-mediated inflammation in neoplastic areas, sharing a common response with neurodegenerative disorders.

In these brain diseases, astrocyte reactivity is a clear hallmark of glia-mediated inflammation and is a critical contributor to the local inflammatory environment of the brain parenchyma. Specifically, these reactive morphological changes, together with the anatomical location of protoplasmic astrocytes, are thought to be important factors for the regulation of blood cell recruitment (Voskuhl et al., 2009), particularly the infiltration of T lymphocytes, which is an essential event in immune responses in the CNS.

The frequency of T-cell entry into the brain parenchyma is low in a healthy brain. Limited subsets of T-cells patrol the CNS tissue, carrying out regular immuno-surveillance. Thus, a massive migration of immune cells to the CNS takes place only during neuro-inflammation (Engelhardt and Ransohoff, 2012). The significance of T-cell infiltration is critical in many CNS inflammatory responses. T-cells are involved in the destruction of myelin sheaths in multiple sclerosis and in experimental allergic encephalomyelitis. Additionally, brain-infiltrating T-cells are crucial to controlling brain infections, as occurs in viral or bacterial invasion and in other cases of encephalitis. However, the function of CNS-infiltrating T-cells in other brain diseases, such as in brain cancer or in neurodegenerative diseases, remains poorly understood. It is thought that certain specific subpopulations of T-cells, such as regulatory T-cells, may facilitate the progression of brain tumors (Sugihara et al., 2009); these T-cells are putative targets for immunotherapy (Von Boehmer and Daniel, 2013). Degenerative diseases, such as Alzheimer's disease and Parkinson's disease, also show infiltrating T-cells in degenerating brain regions, but the role of these infiltrating T-cells remains unclear (Togo et al., 2002; Brochard et al., 2009).

Astrocyte reactivity, and the subsequent entry of T-cells to the brain parenchyma, may be a fundamental aspect of the inflammatory response in CNS diseases. Thus, technical approaches for imaging the inter-relationship between T-cells and reactive astrocytes *in vivo* in the neuro-inflammatory environment are crucial to understanding the intricate phenomenon of T-cell infiltration and its function.

## How to visualize astrocytes in the tissue

To visualize astrocytes in tissue, the use of GFAP-specific antibodies for immunohistochemistry techniques results in specific, feasible and reliable staining. GFAP immunohistochemistry is particularly suitable for mature fibrous astrocytes and reactive astrocytes, although the levels of GFAP are heterogeneous in astrocytes, and GFAP is also expressed in progenitor cells in the adult mouse (Garcia et al., 2004). Other markers, such as S100B, Reelin, and vimentin, have the limitation of identifying other differentiated cell types, such as oligodendrocytes or neurons, making it difficult to distinguish astrocytes from other mature cells (Molofsky et al., 2012). Antibodies against S100B, a glia-specific calcium binding protein, provide robust astrocytic detection but also label mature oligodendrocytes. The use of antibodies against Reelin/Slit, an extracellular matrix protein, detects astrocytes in the early stages of development but may also label neurons. Antibodies against vimentin, which strongly label reactive astrocytes, may also label amoeboid microglia and active macrophages [for an extended list of astrocytic markers, see the article by Molofsky et al. (2012)]. Because GFAP-specific antibodies do not bind to other differentiated cell types, this marker is most likely the best available option for in tissue studies. By contrast, one of the disadvantages of GFAP immunohistochemical staining in tissue is that GFAP does not identify the entire cell body; additionally, some of the micro-anatomical characteristics and details of astrocytes are not easy to visualize under the microscope. Studies performed using transgenic mice with enhanced GFP (eGFP)-expressing astrocytes (Nolte et al., 2001; Suzuki et al., 2003) allowed the imaging of entire astrocytes in a living brain. High-resolution imaging of eGFP-expressing astrocytes reveals fine processes emerging from the cell body, whereas GFAP immune-reactivity remains limited to the perinuclear areas and the thick processes (Suzuki et al., 2003). This result advocates the use of eGFP as preferable, when possible, because eGFP provides detailed morphological information about the entire cell that cannot be detected with GFAP immunohistochemistry. Another option that allows a fine and detailed analysis of the entire astrocytic cell is the dye-filling method, which has the advantage of inoculating specific dyes within fixed brain tissue after extraction and fixation (Wilhelmsson et al., 2006); thus, this technique can be used in fixed tissue from human biopsies.

Currently, the two best microscopy options for visualizing brain cells within tissue are confocal and two-photon microscopy. Both techniques are complementary and can be used to answer different questions regarding the visualization of astrocytes. Two-photon microscopy allows the study of live cells *in vivo* within the brain (Theer et al., 2003; Helmchen and Denk, 2005). With this approach, live cells can be visualized several hundred microns deep within the tissue of living animals, and this approach has the advantage that the interactions of living cells can be studied in time lapse experiments (Theer et al., 2003; Helmchen and Denk, 2005). However, particularly deep brain areas, such as the basal ganglia, thalamus, and other associated structures, are difficult, if not impossible, to visualize unless micro-endoscopy is used (Jung et al., 2004). However, the resolution of two-photon microscopy is still insufficient to visualize the micro-anatomical details of intercellular interactions; furthermore, the availability of important fluorophores prevents the labeling of multiple structures or molecules simultaneously *in vivo*. Although confocal analysis of fixed cell culture preparations or of fixed tissue slides provides static images, the advantage of this analysis is the ability to observe structures at high resolution; thus, the micro-anatomical details of intercellular interactions can be appreciated. In addition, with multiple fluorochromes available, the use of confocal microscopy allows the use of many markers simultaneously, permitting the visualization of several structures with high detail in the same frame. For these reasons, confocal microscopy in fixed tissue may be preferable for analyzing the micro-anatomical details of intercellular interactions at high-resolution, whereas intra-vital two-photon microscopy is preferable for studying the dynamic view of the cellular interactions in the brain parenchyma.

To successfully study the micro-anatomical details of intercellular interactions in three-dimensional space within fixed tissue, the immunohistochemistry protocol requires the following technical specifications: (1) thick sections of tissue for recording the maximum amount of information; (2) specific antigen retrieval treatment for particular epitopes; and (3) long incubations of 24–48 h with primary antibodies. These specifications allow the antibodies to optimally penetrate to 50–60 μm within the tissue, obtaining uniform staining throughout the thickness of the sections. Therefore, the confocal analysis can be performed in depth, and the interactions between the cells can be visualized and analyzed at high detail. In addition, confocal scanning requires a large number of optical sections in order to record high-resolution detail along the z-axis, spanning entire cells and the surrounding interactions, giving the possibility of rendering stacks of images in 3-dimensional space with the appropriate blending software. When scanning is performed, 3-dimensional analyses are important for accurate interpretation of the data and quantifications. This technological approach allows the adequate study of intercellular communication at micro-anatomical levels to understand the intricacy of the glia-mediated inflammatory response in the brain. Based on these technical specifications and on the abridged literature, in the following sections, we review the micro-anatomical details of astrocyte–T-cell interactions in immune-mediated inflammation.

## Imaging astrocytes as chemokine producers and lymphocyte attractants

Astrocytes are important players in the formation of the BBB. Protoplasmic astrocytes, residents of the gray matter, are intimately associated with BVs and, together with pericytes and endothelial cells, control blood flow and BBB permeability (Attwell et al., 2010). In the perivascular compartment, astrocytes are able to produce and release certain proteins that contribute to the neuro-inflammatory response in the CNS (Croitoru-Lamoury et al., 2003). Among these proteins, chemokines are some of the most prominent factors involved in the induction of the pro-inflammatory environment in tissues (Luster, 1998). Chemokines are small proteins that are released into the parenchyma or into the blood stream, creating a gradient of chemo-attractants that other cells follow toward the chemokine source (Luster, 1998; Murdoch and Finn, 2000). Dendritic cells, neutrophils, and monocytes, among others, are typical cellular sources of chemokines in most tissues (Scapini et al., 2000; Zlotnik and Yoshie, 2000). However, in the brain, astrocytes appear to assume a significant portion of this role. *In vitro*, astrocytes respond to inflammatory stimuli, such as bacterial lipopolysaccharide (LPS) and pathogens, by expressing CCL and CXCL chemokines. After LPS treatment to simulate inflammation *in vitro*, a strong increase in the release of CCL2 (MCP-1), CCL3 (MIP-1α), CCL5 (RANTES), CXCL1, and CXCL2 is observed from astrocytes (Van Neerven et al., 2010). Other inflammatory stimuli, such as the treatment of astrocytes with TNF-alpha *in vitro*, also result in the increased expression of several genes in astrocytes, including those encoding the chemokines CCL2, CCL5, and CXCL8 (IL-8) (Meeuwsen et al., 2003). Importantly, in various *in vivo* models of neurodegenerative diseases, the expression of these chemokines is increased specifically in astrocytes. In an experimental model of multiple sclerosis, astrocytes are also responsible for the release of CCL2, CCL3, and CCL5 (Quinones et al., 2008). Consistent with this observation, astrocytes were also found to be the predominant source of CCL2 and CCL3 chemokines in the striatum and the substantia nigra in an experimental model of Parkinson's disease induced by MPTP (Kalkonde et al., 2007). In addition, in an experimental model of Alzheimer's disease, beta-amyloid was shown to activate astrocytes to produce CCL2 and CCL5 (Johnstone et al., 1999). Furthermore, in other scenarios, such as mechanical injury to the brain, chemokines such as CCL2 are also expressed by astrocytes (Glabinski et al., 1996).

**Figure 2:**
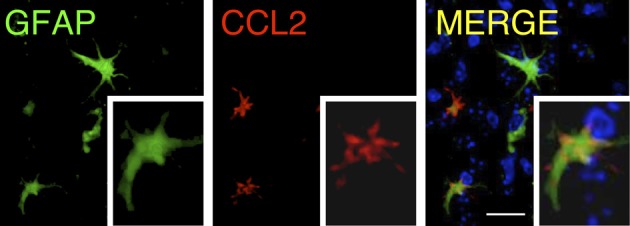
**Distribution of CCL2 in GFAP^+^ astrocytes.** Confocal rendering of an area of LPS-induced inflammation in the mouse brain. Astrocytes are labeled using immunohistochemistry with antibodies against GFAP (green), and the chemokine CCL2 is stained with anti-CCL2 antibodies (red). Nuclei are counterstained with DAPI (blue). CCL2 is expressed in astrocytes, and its intracellular distribution is higher in the terminal GFAP filaments [modified from Carrillo-de Sauvage et al. (2012)]. Scale bar: 30 μm.

**Figure 3:**
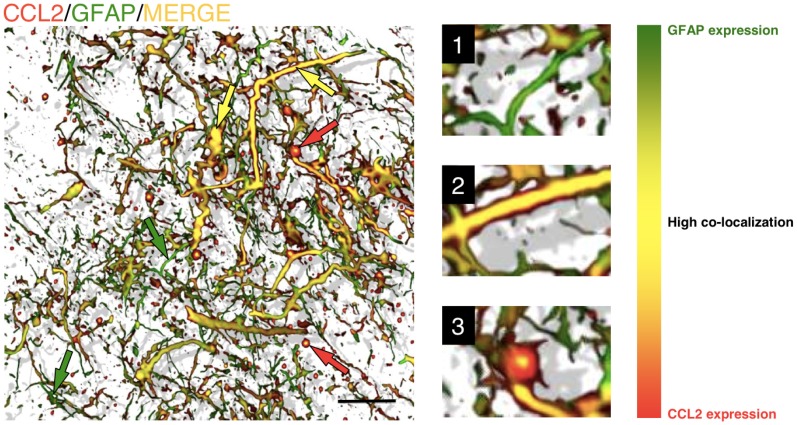
**Astrocytes in GBM are able to adopt various shapes and differentially express chemokines.** Three-dimensional reconstruction, rendered from confocal images, of GFAP/CCL2^+^ astrocytes within a human brain tumor section (biopsy from glioblastoma). It is not possible to distinguish tumorigenic astrocytes from resident astrocytes, but cells adopt different shapes in the neoplastic areas, ranging from star-shaped cells to elongated cell bodies with thick and long filaments. The level of co-localization between GFAP and CCL2 can be observed as a color gradient. This gradient is shown in the scale on the right. Smaller GFAP^+^ cells and thinner filaments are shown in green, with low levels of CCL2 expression (green arrows and detail in 1). Larger cells and thick filaments are shown in yellow, which represents high co-localization of both markers (yellow arrows and detail in 2), suggesting that the expression of CCL2 is highly expressed in reactive astrocytes. Occasionally, astrocytes also show gem-like buds with large accumulations of CCL2 (red arrows and detail in 3). These accumulations, with high CCL2 expression and low GFAP expression, are related to the infiltration of T-cells into the tumorigenic areas [more information about this finding is presented in Carrillo-de Sauvage et al. (2012)]. Scale bar: 20 μm.

These findings indicate that, among the chemokines, CCL2 release is a common factor that is important and predominant in areas of inflammation in the CNS. The mechanism by which astrocytes are activated and produce CCL2 in brain trauma or neuro-inflammation is not well-defined, but it is thought to be associated with stimulation by ATP released at injured areas, which activates P2X7 receptors in the astrocyte membranes (Panenka et al., 2001). After injury, CCL2 is expressed by protoplasmic reactive astrocytes under pro-inflammatory conditions. At the cellular level, CCL2 expression, detected by immunoreactivity, is associated with GFAP, although the intracellular co-localization of both proteins is not complete (Carrillo-de Sauvage et al., 2012). CCL2 immunoreactivity seems to be particularly associated with the terminal tips of GFAP filaments (Figure 2), which suggests a specific role of CCL2 at the edge of astrocyte filaments. In pro-inflammatory conditions in the brain, such as following LPS treatment or viral infection, GFAP^+^CCL2^+^ astrocytes have the characteristic astrocytic star shape. However, particularly in brain tumors, GFAP^+^CCL2^+^ cells adopt convoluted shapes, and CCL2 expression displays different, irregular patterns (Figure 3), depending on the anatomical complexity of the tumor. In all three pro-inflammatory scenarios, these CCL2-rich terminals are specifically related to the neurovascular unit, suggesting that CCL2 may facilitate the entry of blood cells into the brain parenchyma (Carrillo-de Sauvage et al., 2012).

**Figure 4:**
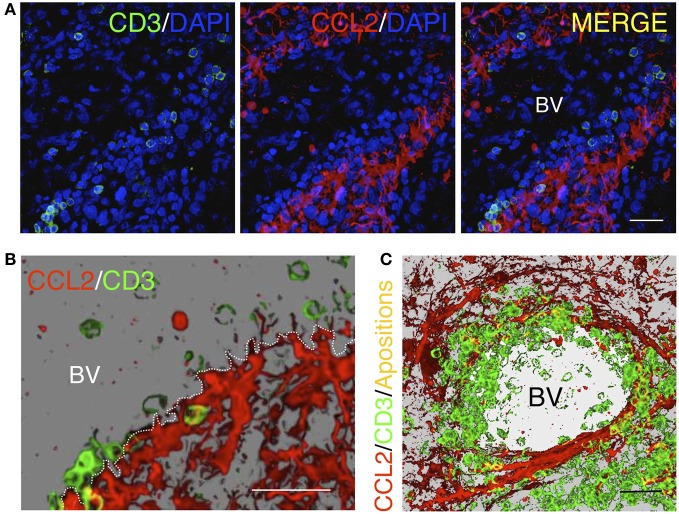
**Homing of T-cells in a tumorigenic area in the human brain. (A)** Confocal images of immunofluorescence for CD3^+^ T-cells (green) and the chemokine CCL2 (red). Cell nuclei are counterstained with DAPI (blue). CCL2^+^ astrocytes delimit the outer perivascular area of the BV, whereas T-cells are localized near CCL2^+^ perivascular astrocytes. **(B)** Three dimensional reconstruction of a detailed region obtained from the confocal images shown in **(A)**. **(C)** Three dimensional reconstruction of a blood vessel lumen in a human glioma. Note that T-cells are apposed to the perivascular CCL2^+^ cells covering the internal wall of the BV lumen. Yellow spots represent co-localization of CD3 and CCL2, representing the areas of contact between the two cell types [Modified from Carrillo-de Sauvage et al. (2012)]. Scale bar: 40 μm in **(A)** and **(C)**; 50 μm in **(B)**.

Chemokines such as CCL3 and CCL4 mainly function as macrophage chemo-attractants (Maurer and Von Stebut, 2004), whereas CCL5 is known to predominantly function in the selective infiltration of T-cells (Schall et al., 1990; Kawai et al., 1999). However, it is not clear which mechanisms may drive the differential entry of particular subsets of cells. For example, CD45RA^−^ CD45R0^+^ memory T-cells migrate in response to the combination of CCL5, CCL2, and CCL3 chemokines (Qin et al., 1996), which indicates that a particular combination of chemokines may stimulate the recruitment of specific T-cell subsets. We and others have recently shown that CCL2 has a primary role in T-cell infiltration in inflamed tissues, particularly in tumors (Brown et al., 2007; Molon et al., 2011; Carrillo-de Sauvage et al., 2012). However, further research must be performed to clarify the role of chemokines in the infiltration of specific blood cell types and to determine whether this response is different in particular anatomical compartments or in specific inflammatory scenarios. Consistent with this finding, one of the crucial factors that induces the differential entry of blood cells is the presence of specific chemokine receptors on the surface of blood cells that signal the activation of diapedesis and parenchymal entry. In this context, particular chemokines, such as CCL2, are able to induce the formation of T-cell uropods, which are micro-anatomical structures that facilitate extravasation (Del Pozo et al., 1997). Importantly, the recruitment of T-cells into the brain occurs at the border of BVs where CCL2^+^ astrocytes are present (Carrillo-de Sauvage et al., 2012). In the BV border, multiple physical contacts between T-cells and CCL2^+^ astrocytes can be observed, particularly surrounding BVs and in areas of inflammation, which suggests the involvement of a micro-anatomical interaction with perivascular astrocytes in T-cell diapedesis (Figure 4). These events are observed in LPS-induced inflammation in mice, in adenoviral-injected monkeys and in astrocytomas in human brains (Carrillo-de Sauvage et al., 2012), suggesting that astrocytes located at perivascular areas may play fundamental chemical and physical roles in recruiting particular T-cell subpopulations into areas of brain inflammation.

Further studies are required to fully understand the role of astrocytes in the infiltration of T-cells and the mechanism underlying the differential entry of specific subpopulations of lymphocytes into the inflamed brain. Most likely, astrocytes release different types of chemokines depending on the inflammatory milieu, creating a route for specific lymphocyte populations to follow. Importantly, disentangling this information *in vivo* will have crucial implications for therapeutic strategies for brain diseases in the near future.

## Imaging the mutual arrangement in cell-to-cell immunological encounters

The interaction between astrocytes and lymphocytes in immune-mediated inflammation is not a static event. Both cell types undergo different changes in the process of approaching, apposing and contacting each other. As we described in the previous section, lymphocytes and astrocytes signal to each other to establish a contact that may result in the entry of lymphocytes into the brain parenchyma. However, other types of lymphocyte–astrocyte contacts that occur in the tissue during immune-mediated antigen presentation may also help us to understand the features of cell-to-cell arrangements that control brain immune responses.

**Figure 5:**
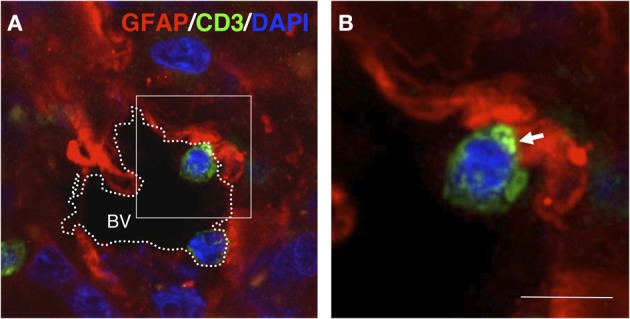
**T-cell–GFAP interactions in gliomas.** Confocal section (0.5 μm) obtained from a human glioma biopsy. Immunohistochemistry was performed with anti-GFAP antibodies (red), anti-CD3 antibodies (green) for T-cells and DAPI for counterstaining (blue). **(A)** Overview of a blood vessel (BV) in the tumor area in which two T-cells are contacting GFAP^+^ cells at the edge of the vascular lumen. **(B)** High magnification of the frame indicated in **(A)** showing details of the T-cell–GFAP interaction. Importantly, the apposed T-cell displays clustered CD3 at the interface of the intercellular contact (arrow) [original findings can be found in Barcia et al. (2009)]. Scale bar: 15 μm.

T-cells form a complex structure called an immunological synapse (IS) when contacting antigen-presenting cells (APCs) (Dustin, 2005). At the level of the T-cell–APC interface, this micro-anatomical structure is characterized by the specific arrangement of several adhesion molecules and receptors, such as lymphocyte function-associated antigen (LFA)-1, CD3, and the T-cell receptor (TCR), as well as the reorganization of organelles such as the microtubule organizing center (MTOC), the Golgi apparatus, and the F-actin and alpha-tubulin cytoskeleton (Kupfer et al., 1983; Monks et al., 1998; Stinchcombe et al., 2006). Most of the studies on ISs have focused on the changes that T-cells undergo, but little attention has been given to APC transformations. The first visualization of the IS *in vivo* was made in T-cells contacting adenovirus-infected astrocytes in the brain (Barcia et al., 2006). The arrangement of the T-cells *in vivo* was similar to the micro-anatomical organization previously described *in vitro* (Monks et al., 1998). The arrangement of T-cells mainly involves the clustering and polarization of the TCR/CD3 complex at the center of the intercellular interface and the segregation of LFA-1 to the outer ring of the interface (Monks et al., 1998; Dustin, 2005). The role of this arrangement at the IS remains unclear. The TCR/CD3 complex is particularly important in the detection of antigen and in the subsequent signaling that activates the T-cell. In addition, the LFA-1 arrangement is crucial in adhesion and cytoskeletal reorganization. LFA-1 is linked to TALIN and to the cytoskeleton, which guides the polarity of the T-cell after adhesion. These arrangements occur in T-cells in many immune-mediated scenarios. In the brain, T-cells rearrange their interface, forming typical mature ISs, when contacting viral-infected astrocytes or glioma cells (Figure 5) (Barcia et al., 2006, 2009; Yang et al., 2010). It is thought that this complex arrangement of the T-cell interface is important for a directional effector response, but further studies are required to unravel its function. T-cell–APC contact may result in the elimination of the target cell because of the release of cytotoxic granules, such as IFN-gamma, granzyme-B, or perforin, by the T-cell at the synaptic interface. In this context, targeted APCs undergo different morphological and biochemical changes that lead to the APCs final elimination. T-cell IS formation appears to be fundamental for the clearance of astrocytes infected with adenovirus from the brain (Barcia et al., 2006). In this context, IFN-gamma is polarized in T-cells at the IS established with adenovirus-infected astrocytes; thus, IFN-gamma is specifically delivered toward the synaptic interface for an effective immune response (Barcia et al., 2008b). In the case of glioma cell clearance, granzyme-B is polarized in cytolytic T-cells and delivered to the synaptic interface toward the target glioma cell. Granzyme-B induces the specific cleavage of alpha tubulin (Adrain et al., 2006), disrupting the glioma cell cytoskeleton (Barcia et al., 2009), and leading to CTL-mediated apoptosis of the target cells (Goping et al., 2006).

**Figure 6:**
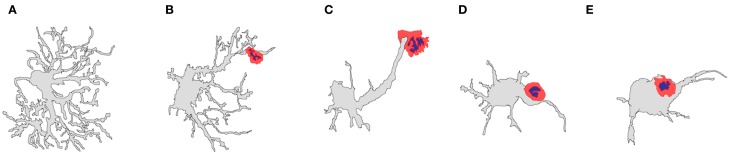
**Diagrams representing the shape variation that can be found in T-cell–astrocyte interactions in the rat striatum.** Adenovirus-infected astrocytes display different shapes as the T-cell apposition become closer to the astrocyte cell body. **(A)** Typical astrocytic shape in the brain; **(B)** an astrocyte in contact with a T-cell lymphocyte (red) at its terminal processes; **(C)** an astrocyte in contact with a T-cell lymphocyte (red) distally at the astrocytic protrusion; **(D)** an astrocyte in contact with a T-cell lymphocyte (red) proximally at the astrocytic protrusion; **(E)** an astrocyte in contact with a T-cell lymphocyte (red) at the cell body [artistic sketches performed by CB, modified from Barcia et al. (2008a)].

**Figure 7:**
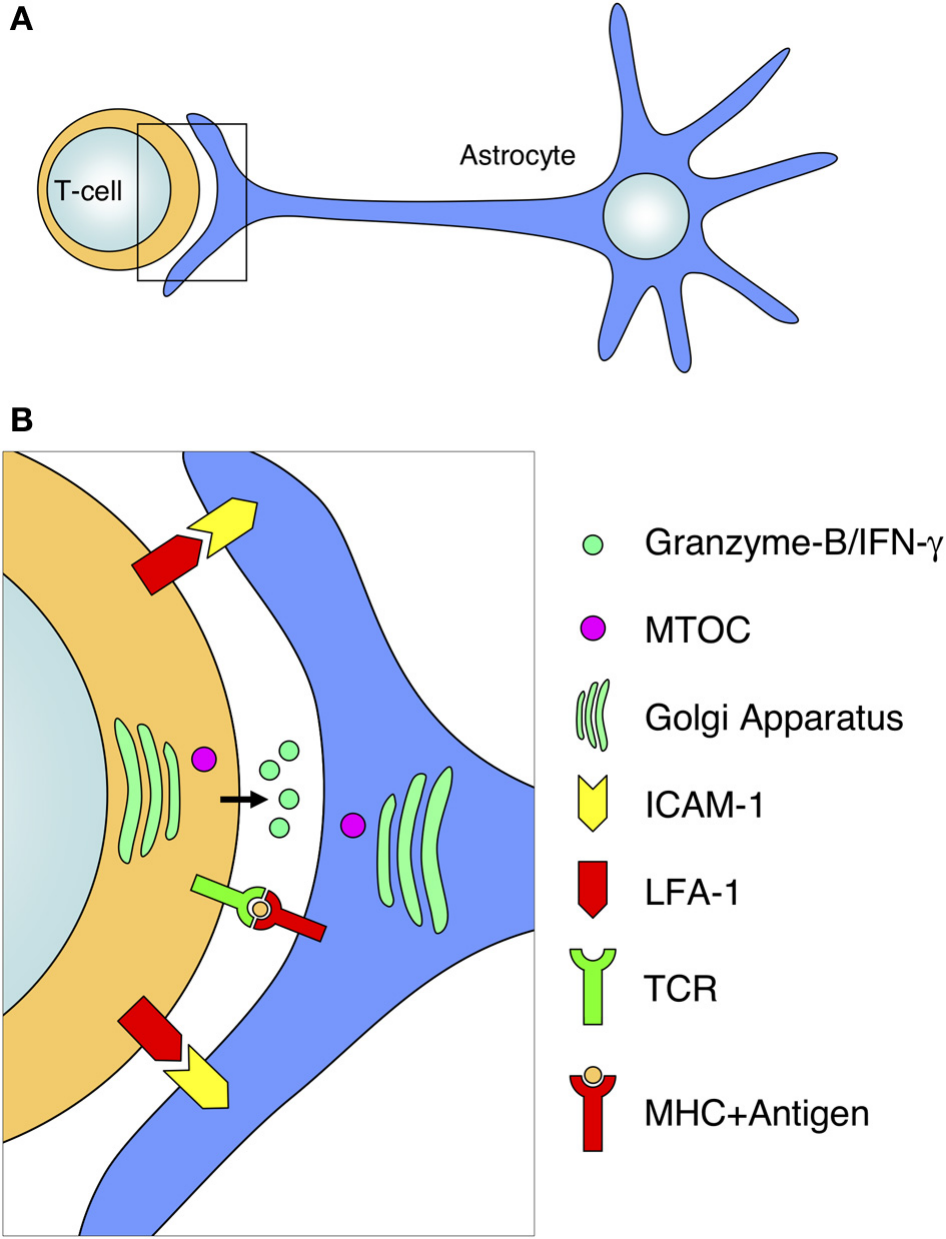
**Diagram of antigen-dependent T-cell–astrocyte interactions.** The interaction of activated T-cells with MHC-expressing astrocytes involves a defined arrangement of various molecules and organelles. **(A)** Schematic representation of the interaction of a T-cell with an astrocyte at the level of the astrocytic protrusion. **(B)** Magnification of the T-cell–astrocyte interface from the frame depicted in **(A)**. The T-cell–astrocyte interface is characterized by the segregation of adhesion molecules (such as LFA-1 and ICAM-1), the interaction of the TCR with MHC molecules, the release of cytotoxic compounds (such as Granzyme-B or IFN-gamma) into the intercellular space; and the rearrangement and polarization of organelles toward the interface (for example, the relocation of the MTOC and Golgi apparatus in both cell types).

Notably, target astrocytes in intimate immunological contact with T-cells also have similar molecular and micro-anatomical arrangements as those in lymphocytes. Astrocytes, functioning as APCs, establish ISs with T-cells and undergo massive morphological changes characterized by a reduction in cell body size, a reduction in the number of filaments, and the formation of a protrusion toward the area of contact (Figure 6) (Barcia et al., 2008a). Importantly, the MTOC and the Golgi apparatus also re-orient toward the interface, frequently contacting the astrocytic protrusion, suggesting that astrocytes actively respond to T-cell interactions and are not passive APCs. Similar changes are observed in primary astrocytes in response to a physical lesion; these astrocytes develop a protrusion and reorient their MTOC and Golgi apparatus toward the lesion side (Etienne-Manneville, 2006; Osmani et al., 2006). These results suggest that T-cell apposition signals to the target astrocyte, inducing changes that may result in the interchange of crucial information between the two cells (Figure 7). The function of this symmetric arrangement remains unknown. Astrocytes appear to prepare themselves to release signaling products toward T-cells. In particular, the orientation of the Golgi apparatus and MTOC is consistent with these observations. These changes may be related to the transmission of specific messages such as the confirmation of “kill-me” signals sent by CTLs or, by contrast, the release of “do-not-kill-me” signals to avoid elimination. In addition, because astrocytes have been described to engulf particles or entire cells, it has been hypothesized that this active rearrangement of astrocytes may also represent a physical defense of the targeted astrocyte in the process of engulfing a T-cell aggressor (Barcia et al., 2008a; Loov et al., 2012).

T-cell–astrocyte apposition is the result of the mutual approach of both cells, stimulated by the orchestrated release of cytokines or chemokines into the extra-cellular environment. As described above, particular chemokines released by astrocytes are responsible for the recruitment of T-cell subsets into the brain parenchyma. Conversely, cytokines released by T-cells that have infiltrated into the brain parenchyma interact with local astrocytes and induce specific changes. Particular subtypes of pro-inflammatory cytokines, such as IFN-gamma, stimulate the synthesis and consequent expression of MHC molecules on the astrocytic membrane, and these molecules are required for the presentation of antigens to T-cells. Indeed, MHC-expressing astrocytes of the CNS are efficient APCs that are able to strongly activate antigen-reactive T-lymphocytes (Fierz et al., 1985; Wekerle et al., 1987). In particular, in the context of the adaptive immune response in the brain, MHC expression by astrocytes is highly upregulated by IFN-gamma, which is released by activated T-cells that infiltrate the brain parenchyma (Richt et al., 1990; Barcia et al., 2008b). Notably, a detailed micro-anatomical analysis of MHC expression in astrocytes revealed that MHC molecules accumulate at a high density in the cell body and the proximal areas, in contrast to the low levels that accumulate in the distal areas, such as in filaments or protrusions (Barcia et al., 2008a). These findings suggest that the astrocytic cell body has a gradient of affinity for T-cells. This strategy of approach and final contact suggests that astrocytes may have an active and pivotal role in the development of immune responses in the CNS, acting as APCs in response to cytokines (Fierz et al., 1985). Thus, T-cell–astrocyte interactions in brain immune responses may contribute to different outcomes depending on the released factors. A key element in this modulation is the fact that specific chemokines or cytokines induce particular T-cell subpopulation responses and may determine the type of immune response. Through different mechanisms, astrocytes are able to both prevent or promote the activation of T-cells (Wekerle et al., 1987). One of the strategies to suppress T-cell activation is the upregulation of CTLA-4, which inhibits Th1 and Th2 cell responses occurring with or without astrocyte–T-cell contact (Gimsa et al., 2004). Another strategy is the release of IL-10, which directly activates regulatory T-cells (T_regs_). Both strategies are able to attenuate cytolytic immune responses in the brain, which may be beneficial for reducing the aggressive autoimmune responses that occur in multiple sclerosis or experimental autoimmune encephalomyelitis. By contrast, these approaches also increase tolerance, which may be advantageous for tumor cell growth or may facilitate the expansion of infections and the development of encephalitis. Astrocytes are also able to promote the activation of T-cells through the release of certain cytokines by alternative mechanisms. In autoimmune diseases, astrocytes release IL-15, which contributes to an increase in T-cell effector function (Saikali et al., 2010), or IL-17, which activates cytolytic immune responses, facilitating cephalitogenic responses to local CNS inflammation, such as in multiple sclerosis.

Thus, the stimulation or inhibition of cytolytic or regulatory arms through the release of chemokines or cytokines by atrocytes may result in markedly different outcomes that modulate immune responses in the brain. Targeting cytokines/chemokines or their receptors can alter the course of several neurological diseases, and the effects may be beneficial or harmful, depending on the particular situation (Steinman, 2013). Therefore, the study of the mechanisms underlying T-cell–astrocyte communication will be important for the design of therapeutic strategies for neurodegenerative disorders and brain tumors that show T-cell infiltration.

## Concluding remarks

Little is known about intercellular communication in immune-mediated responses in the brain. Specifically, the role of astrocytes in adaptive and innate responses has scarcely been explored. Evidence suggests that protoplasmic astrocytes may have important functions in the regulation of immune responses in the brain, particularly regarding the infiltration of lymphocytes and their subsequent interactions with APCs once inside the brain parenchyma.

Many brain disorders, including brain cancer, involve lymphocyte infiltration and astrocyte reactivity; nevertheless, the interactions between these cell types have been largely overlooked. This large area of research remains to be explored and will produce extremely valuable information for treating CNS diseases. Astrocyte–lymphocyte communication could be targeted for therapeutic purposes in the near future.

### Conflict of interest statement

The authors declare that the research was conducted in the absence of any commercial or financial relationships that could be construed as a potential conflict of interest.
